# The Difficulty in Numerical Computation Impacts Motor Decisions in a Stop‐Signal Task

**DOI:** 10.1111/ejn.70410

**Published:** 2026-02-03

**Authors:** Isabel Beatrice Marc, Valentina Giuffrida, Mariella Segreti, Ann Paul, Sabrina Fagioli, Pierpaolo Pani, Stefano Ferraina, Emiliano Brunamonti

**Affiliations:** ^1^ Department of Physiology and Pharmacology Sapienza University Rome Italy; ^2^ Behavioral Neuroscience PhD Program Sapienza University Rome Italy; ^3^ Department of Education University of Roma Tre Rome Italy

**Keywords:** cognitive difficulty, motor decision, numerical distance effect, SST

## Abstract

The proper interpretation of environmental information is necessary for effective decision‐making. The resulting cognitive burden may affect the entire process if interpretation is not instantaneous. In this study, we investigated how numerical distance (ND), a measure of cognitive demand in numerical comparisons, influences movement initiation and inhibition. To this end, 32 participants completed a novel numerical comparison stop‐signal task (NC‐SST), in which the cognitive demand of each trial was manipulated by varying the ND between pairs of numbers presented in both Go and Stop signals. Participants were required to initiate or stop a movement if the number was higher or smaller than the one indicated as reference. Results showed that larger NDs (i.e., easier comparisons) facilitated faster and more accurate responses during movement initiation and enhanced stopping performance. Using a generalized drift‐diffusion model, we found that drift rates increased with Go ND and were modulated by the spatial location of numerical stimuli, consistent with a left‐to‐right space number association. A generalized linear mixed‐effects model further revealed that Go process parameters, particularly the drift rate, strongly predicted successful stopping and interacted with Stop ND and stop‐signal delay (SSD). These findings demonstrate that greater cognitive difficulty impairs both movement initiation and inhibition, and that motor decisions result from the integration of cognitive information onto perceptual features, extending the classical race model framework.

## Graphical abstract

Cognitive difficulty arising from numerical comparison modulates motor control. In a stop‐signal task where the Go and Stop signal corresponded to the result of a numerical comparison, we show that the degree of difficulty in making this mental operation affects both movement initiation and inhibition, highlighting the interaction between cognitive demands and motor decisions.

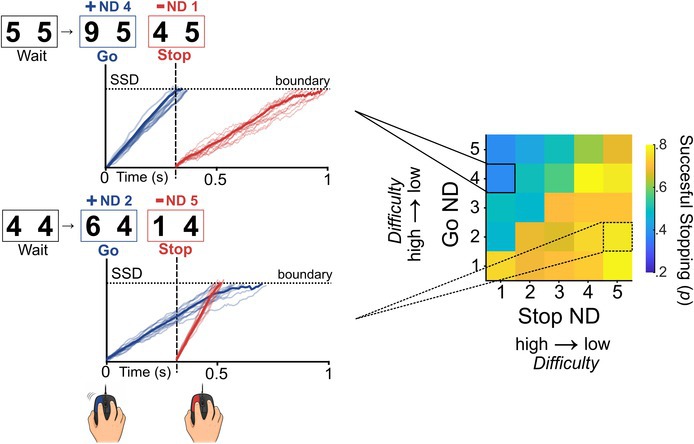

**Abbreviations**
BIC: Bayesian information criterion
cRT: catch reaction time
DDM: drift‐diffusion model
GDDM: generalized drift‐diffusion model
GLMM: generalized linear mixed‐effects model
NC‐SST: numerical comparison stop‐signal task
ND: numerical distance
RT: reaction time
SL: spatial location
SSD: stop‐signal delay
SSRT: stop‐signal reaction time
SST: stop‐signal task

## 1 Introduction

We frequently must decide whether to stop an action in response to unexpected changes in environmental demands. An experimental paradigm that probes this behavior is the stop‐signal task (SST; Logan and Cowan 1984; Aron and Verbruggen 2008; Bissett and Logan 2011). In the SST, participants typically must generate a motor response as quickly as possible in response to a “Go signal” or cancel the planned movement after the presentation of a “Stop signal.” In the context of the SST, the theoretical framework of the “Horse‐Race Model” describes a competition (race) between response inhibition elicited by the Stop signal and response initiation triggered by the Go signal. Within this framework, the winner of the race determines the behavioral outcome, such as whether the response continues or stops. It has been shown that various factors, such as motivation (Leotti and Wager 2010; Boehler et al. 2012, 2014; Giamundo et al. 2021; Giuffrida et al. 2023, 2025; Verbruggen and McLaren 2018), attention (Verbruggen et al. 2014; Hilt and Cardellicchio 2020; Haque et al. 2024), and the perceptual characteristics of the stimuli (Montanari et al. 2017; Middlebrooks et al. 2019; Pani et al. 2018; Marc et al. 2023), can influence performance when canceling the planned movement is required.

The influence of cognitive difficulty on motor control during visuomotor transformation has been less thoroughly investigated. Some experimental contexts have explored this decision difficulty using tasks that require comparisons between rank‐ordered items and numerical pairs (Merritt and Terrace 2011; Brunamonti et al. 2012, 2016; Jensen et al. 2017; Mione et al. 2020). These experiments have demonstrated that the difference between the ranks or between the numerical values of the stimuli influences decision‐making accuracy and latency (see Ramawat et al. 2023, for a review).

A mechanistic account for these difficulty effects is provided by spatial‐numerical associations. In this framework, numerical magnitudes are spatially oriented from left to right with single positions partially overlapping (Dehaene et al. 1993; Dehaene 1997; Hubbard et al. 2005; Umiltà et al. 2009, see Fischer and Shaki 2014 for a review). According to studies demonstrating an association between numerical and spatial information, one determines whether 7 is greater than 5 by comparing their positions on this internal mental representation, where small numbers (e.g., 1) are associated with the left side of the space and larger numbers (e.g., 9) with the right (Longo and Lourenco 2007). A phenomenon observed during numerical comparisons is the numerical distance (ND) effect, which modulates accuracy and reaction time depending on the distance in the internal representation (Moyer and Landauer 1967; Hubbard et al. 2008). When two numbers are relatively far apart, participants respond faster and more accurately than when they are closer (Izard and Dehaene 2008; Holloway and Ansari 2008).

The study investigates the effect of the ND on motor control, with the underlying hypothesis that assigning a cognitive difficulty onto perceptual detection modulates motor decisions. Here, we developed a numerical comparison stop‐signal task (NC‐SST), where participants evaluated numerical pairs, which integrate ND into both Go and Stop signals. The behavioural results demonstrated that ND, manipulated across five levels, had a significant effect not only on movement initiation but also on inhibition, reflecting the interplay between cognitive difficulty and motor control. With this design, we could also test whether differences in cognitive difficulty would be reflected in the underlying dynamics of decision‐making processes. We used the drift‐diffusion model (DDM; Ratcliff 1978; Shinn et al. 2020) for this purpose, which views decision‐making as the process of accumulation of information over time. Based on this framework, we hypothesize that larger ND, reflecting easier comparisons, would be associated with faster drift rates, leading to quicker response initiation. To further examine how the accumulation of Go‐related evidence influences movement inhibition, we applied a generalized linear mixed‐effects model (GLMM) to predict stopping success on a trial‐by‐trial basis. This analysis tested whether faster Go drifts impaired the ability to stop, and whether this effect was modulated by the characteristics of the Stop signal, such as its ND from the Go signal, as supported by our results.

## 2 Methods

### 2.1 Participants

Thirty‐two healthy participants (27 females and 5 males, mean age 25 ± 4.4) were recruited for the study. According to the Edinburgh Handedness Inventory (Oldfield 1971), all participants were tested for manual dominance, and there were 1 left‐handed and 31 right‐handed participants. Participants had normal or corrected vision without any known neurological or psychiatric conditions. All procedures followed the Declaration of Helsinki, and after obtaining written informed consent from each participant. The procedure was approved by the Ethics Committee of “Roma Tre” University.

### 2.2 Experimental Procedure and the NC‐SST

**FIGURE 1:**
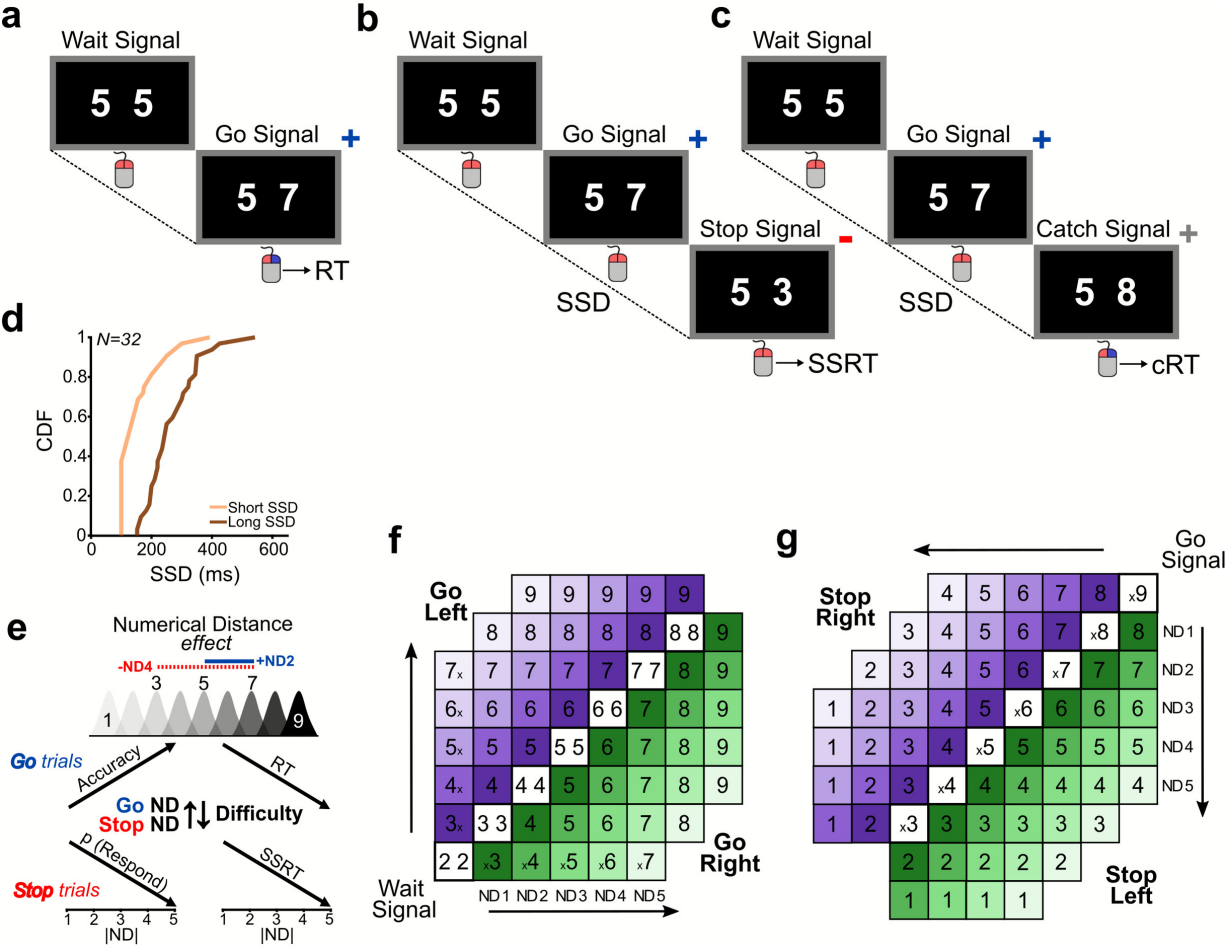
**Illustration of the experimental task and the manipulation of the degree of difficulty in the different conditions. (a–c)** Time course of the task's three different trial types. **(a)** In Go trials, the mouse button that needed to be released was indicated by an increasing value of the number on one side of the screen (Go signal). **(b)** In Stop trials, a decrease in the value at the same screen position following the Go signal indicated to stop the engaged movement. **(c)** In Catch trials, a second increase in value indicated to continue and complete the movement. **(d)** The cumulative distribution function (CDF) displaying participants' SSD distributions (*N* = 32; short SSD = light orange; long SSD = brown). **(e)** The hypothesized spatial representation of the number's hierarchy. The ND effect is illustrated through distributions, where lower NDs cause greater overlap in mental representation, which makes comparisons harder, while higher NDs reduce overlap, which makes comparisons easier. Hypothesized behavioral outputs in Go and Stop trials. In Go trials, accuracy is increasing while RTs are decreasing, while in Stop trials, p(Respond) to the Stop Signal and SSRT are decreasing with greater ND. **(f, g)** Schematic representation of all combinations in Go and Stop trials. **(f)** All Wait Signals are presented in the diagonal squares, same number on both sides of the screen. The following Go signal was represented by an increase in the value of one of the two numbers, either on the Left (N x, purple color scales) or on the Right (x N; green color scales) as a function of NDs. (**g)** In Stop trials, diagonal squares represent the Go signals, and the following Stop signal was represented by a decrease in the value as a function of ND from the Go signal. cRT, catch reaction time; ND, numerical distance; RT, Go reaction time; SSD, stop‐signal delay; SSRT, stop‐signal reaction time.

Participants were seated in a darkened, sound‐attenuated room facing a monitor (15.6 in., 1920 × 1080 resolution, refresh rate 60 Hz) on a laptop computer that was comfortably situated 50–60 cm away. Under different experimental conditions, participants performed a modified version of the SST, in which the Go and the Stop signals were represented by the outcome of a mental computation, rather than the encoding of a perceptual stimulus. Each participant held a mouse while resting their arm on a table. Each trial started when both effectors (the index and middle fingers) simultaneously pressed both mouse buttons to reach their initial positions. Every time the buttons were pressed, a Wait signal would show two identical numbers, such as 5‐5, 283 pixels to the left and right of the center of the screen. Each of the numbers was 20 × 28 pixels in size and white, and the overall resolution of each image was as high as the screen resolution with a black background (Figure 1a–c). In Go trials (50% of the trials), one of the two numbers increased (for example, 5‐7 from 5‐5) following a variable waiting time (500–1000 ms; in steps of 50 ms).

This change served as the Go signal, which instructed participants to release the mouse button congruent with the spatial location of the number that changed value on the monitor (Figure 1a,f shows all combinations of Wait and Go signals). In Stop trials (25% of the trials), after a variable amount of time (stop‐signal delay [SSD]) from the Go signal, a Stop signal with a smaller value, such as 5‐3 following 5‐7 (Figure 1b,g shows all combinations of Go and Stop signals), was placed after the Go signal. In this case, the Stop signal instructed participants to inhibit the movement triggered by the previous pairs of numbers. To ensure that participants focused on the numerical comparison to be made rather than simply reacting to any changes after the Go signal, we included Catch trials (remaining trials) alongside Stop trials (Figure 1c). In these trials, the stimulus occurring after the Go signal was based on a change to a higher number, such as 5‐8, instructing the participants to continue their movement. These trials served as a control condition and were included in the behavioral analysis to quantify the transient slowing triggered by the Catch signal. First, participants experienced the three trial types (Go, Stop, and Catch) in a familiarization block (~100 trials). During this familiarization block, a tracking procedure dynamically adjusted the duration of the SSD in steps of 50 ms based on the accuracy of the previous Stop trial: The initial value was set to 200 ms, increasing by one step (+50 ms) if the trial was executed correctly and decreasing by one step (−50 ms) if executed incorrectly. Catch trials were presented using the most recently computed SSD value, ensuring consistency with the last adjustment made during the Stop trial. The probability of responding to the Stop during the familiarization block fell within recommended bounds for stable estimation (p(Respond) = 0.56 ± 0.16 StD), satisfying both the 0.40–0.60 target range and the broader 0.25–0.75 limits (Verbruggen et al. 2019) and confirming that the staircase converged appropriately. We then computed the average SSD for each participant during the familiarization block and defined two difficulty conditions based on this value. In line with the horse‐race model, a *short SSD* condition (average SSD − 100 ms; population mean: 161 ± 71 ms) and a *long SSD* condition (average SSD + 50 ms; population mean: 271 ± 86 ms), were taken as easy and difficult conditions, respectively. Figure 1e displays the distribution of these values among participants, which were utilized in the test phase that followed. In addition to the difficulties introduced by the length of the SSDs when interpreting the visual stimuli, as either a Stop or a Catch signal, we varied the ND in the paired number comparison to further manipulate the complexity of cognitive operations needed in the response, from small distances to larger distances (e.g., 4–5: ND1 or 4–9: ND5; for an overview of combinations, see Figure 1f,g). Furthermore, to avoid potential bias brought on by participant expectations, NDs were restricted in Catch trials (using only ND1 or ND2) during the testing phase. This choice ensured that participants could not use the ND displayed on the initial Go signal to anticipate whether a second change would be a Stop or a Catch signal. If Catch signals had appeared across the full ND range, the set of ND values available at the Go signal would necessarily have been constrained, because a Catch signal always requires a larger ND. Consequently, some Go signal NDs would have been more likely to precede a Catch signal than a Stop signal, making the Go signal itself a probabilistic cue. Limiting Catch trials to ND1‐2 attenuated this bias. During this phase, each participant performed the task divided into four blocks with a 10‐min pause between each block (~1500 trials). Two separate auditory feedbacks were used to distinguish between trials that were completed successfully and those that were not. Feedback was delivered using Psychtoolbox's *Beeper* function. Correct responses elicited a single 400‐Hz tone (150‐ms duration), whereas incorrect responses elicited the same tone presented twice, separated by a 200‐ms interval. The presentation of stimuli and collection of behavioral events were handled with MATLAB R2021b (www.mathworks.com), using the Psychophysics Toolbox Version 3 feature set (www.psychtoolbox.org).

### 2.3 Variables Estimation and Data Analysis

The current study aimed to assess how the difficulty of interpreting NDs among numbers influenced both movement initiation and inhibition in response to perceptual signals. To achieve this, we examined how participants' performance was influenced by NDs (from 1 to 5) across both Go and Stop trials. In Go trials, we also assessed the influence of the Spatial Location (SL; Left/Right) of the higher number, while in Stop trials, we evaluated the effect of inhibition timing by manipulating SSDs (Short/Long). Using a 2 × 5 within‐subjects design, we conducted two‐way repeated measures ANOVAs to examine the following: (1) Accuracy, which represents the proportion of correct responses to the Go signal, providing an indicator of overall performance; (2) Go RTs, measured as the time between the presentation of the Go signal and the button release. Omission errors (lack of movement initiation) and commission errors, initiating movement in the wrong SL indicated by the Go signal were excluded; (3) Go RTs Variability, measured as the standard deviation of the Go RT distribution, assessed the consistency of participants' RTs across trials. In addition to these measures of movement initiation, to assess movement inhibition, we also examined (1) p(Respond) to the Stop signal, the probability of responding on Stop trials, which reflected participants' ability to inhibit their response when a Stop Signal was presented; and (2) Stop Signal RT (SSRT), which is a nonparametric estimate of the time participants required to successfully inhibit the response after the Stop signal, computed through the integrative method (Verbruggen et al. 2019). For each ND, this was computed using trials from both SSDs to get an accurate estimate (50 > Stop trials). Each participant's performance was checked for compliance with the independence assumption of the “horse‐race model,” as indicated in Verbruggen et al. (2019) for the reliable estimate of the SSRT. We considered the independence assumption respected if the average Stop Error RTs were shorter than the average Go RTs (De Jong et al. 1995; Verbruggen and Logan 2009). These averages were compared using paired *t*‐tests; the relevant variables were computed for each participant separately and for each ND. Custom MATLAB R2022b (www.mathworks.com) functions were implemented for data processing and analysis.

### 2.4 Fitting the Generalized Drift‐Diffusion and General Linear Mixed‐Effect models

***GDDM*.** Using the PyDDm toolbox (Shinn et al. 2020), we applied the generalized drift‐diffusion model (GDDM) to participants' Go RTs distributions to determine the computational strategies employed across ND. The model was originally developed to describe perceptual decision‐making (Ratcliff and Mckoon 2008), but it has since been expanded to include symbolic manipulation and numerical cognition (Ratcliff et al. 2015; Park and Starns 2015; Krajcsi et al. 2016; Dix and Li 2020). The GDDM conceptualizes decision‐making as a noisy, continuous process in which information accumulates over time, where a choice is made when the decision variable (*dx*) reaches one of two decision boundaries “B,” which in our case correspond to accuracy (correct vs. error) or spatial location of the target choices (left vs. right), based on the model hypothesis tested. To flexibly capture these dynamics, we implemented four models of increasing complexity, each building upon a basic drift‐diffusion framework—the null model. For all the tested models, time resolution (*dt*) was set to 0.01 s, and the overall duration was set to 1.5 s, corresponding to the upper RT limit to respond in Go trials. The null model captures only the basic decision dynamic, not including any influence of the stimulus characteristic or decision context. The evolution of the *dx* is governed by: dx=v*dt+dW.

It assumes a constant drift parameter “v” (= 10), symmetric decision boundaries (= 3) set as “Correct” for upper bound and “Error” for lower bound, a fitted nondecision time (= 0.1 s), and “W” is momentary noise (std = 1).

*Model 1* (*Linear*) builds upon the Null model by adding a condition‐dependent *v* to test whether it varies linearly with *ND*, where the evolution of the *dx* is governed by:

$$\mathrm{dx}=\left[v*\mathrm{ND}\right]*\mathrm{dt}+\mathrm{dW}$$

All parameters are estimated to vary freely within a range (v =−10 to +10, *B* = 0.1 to 3; and nondecision time = 0.05 to 0.4 s).

*Model 2* (*LinearwithLeak*) improves Model 1 by adding the possibility that imperfect integration *leak* of evidence can happen, modeling memory decay, where *dx* is governed by:

$$\mathrm{dx}=\left[v*\mathrm{ND}+\text{leak}*x\right]*\mathrm{dt}+\mathrm{dW}$$

The *leak* parameter (−10 to 10) is a constant that acts as a multiplier, modulating how accumulated evidence *dx* changes over time. A negative leak causes the evidence to decay towards zero, while a positive leak makes it grow exponentially.

*Model 3* (*LinearwithSLbias*) offers an extension to Model 1 by introducing a spatial location bias (SL bias) toward one of the two target choices (Left = upper bound vs. Right = lower bound), spatial biases not accounted for by the ND alone, where *dx* is governed by:

$$\mathrm{dx}=\left[v*\mathrm{ND}+\text{SLbias}\right]*\mathrm{dt}+\mathrm{dW}$$

The SL bias is a free parameter (−5 to +5) capturing a consistent bias toward the Left or Right *B*.

*Model 4* (*LinearwithSLbiasLeak*) integrates upon Models 2 and 3 to offer a more complete description of the decision process. It assumes that the *v* varies linearly with ND, includes SL bias, and adds a *leak* term to capture imperfect evidence integration. The *dx* evolution evolves according to:

$$\mathrm{dx}=\left[v*\mathrm{ND}+\text{SLbias}+\text{leak}*x\right]*\mathrm{dt}+\mathrm{dW}$$

This model provides a flexible framework to test how the ND effect, SL bias, and evidence leak jointly contribute to decision behavior.

***GLMM***. To explore how the processing of Go and Stop signals interacted at the single‐trial level, in a subgroup of 17 participants, in which Go ND information was available, we encoded for each trial the ND eliciting the Stop process, depending on the one that in the same trial triggered the Go process. We then fitted a generalized linear mixed‐effects model (GLMM) to examine how trial‐wise parameters predict successful inhibition. The binary outcome variable S_ij_∈{0,1}, indicating successful (1) or failed (0) inhibition for each trial *i* and participant *j*, was modeled as a function of Go Drift, SSD (Short/Long), Stop ND, and Stop SLbias (Left/Right), along with relevant interactions. The mixed‐effects structure included random intercepts and random slopes for Go Drift by participant to account for individual variability in baseline stopping performance and sensitivity to Go evidence. The model specification:

$$\begin{array}{c} \mathrm{Sij}=\beta 0+\beta 1\text{GoDrift}+\beta 2\text{StopSSD}+\beta 3\text{StopND}+\beta 4\text{StopSLbias}\\ \;+\beta 5\left(\text{GoDrift}*\text{StopSSD}\right) \end{array}$$

$$+\beta 6\left(\text{GoDrift}*\text{StopND}\right)+\beta 7\left(\text{StopND}*\text{StopSSD}\right)+u0j+u1j\;\text{GoDrift}$$

where *β*0 is the intercept, *β*1 to *β*7 are fixed effect coefficients, and *u*_0*j*_, *u*_1*j*_ are participant‐specific random intercepts and slopes.

## 3 Results

### 3.1 Movement Initiation Is Influenced by ND

**FIGURE 2:**
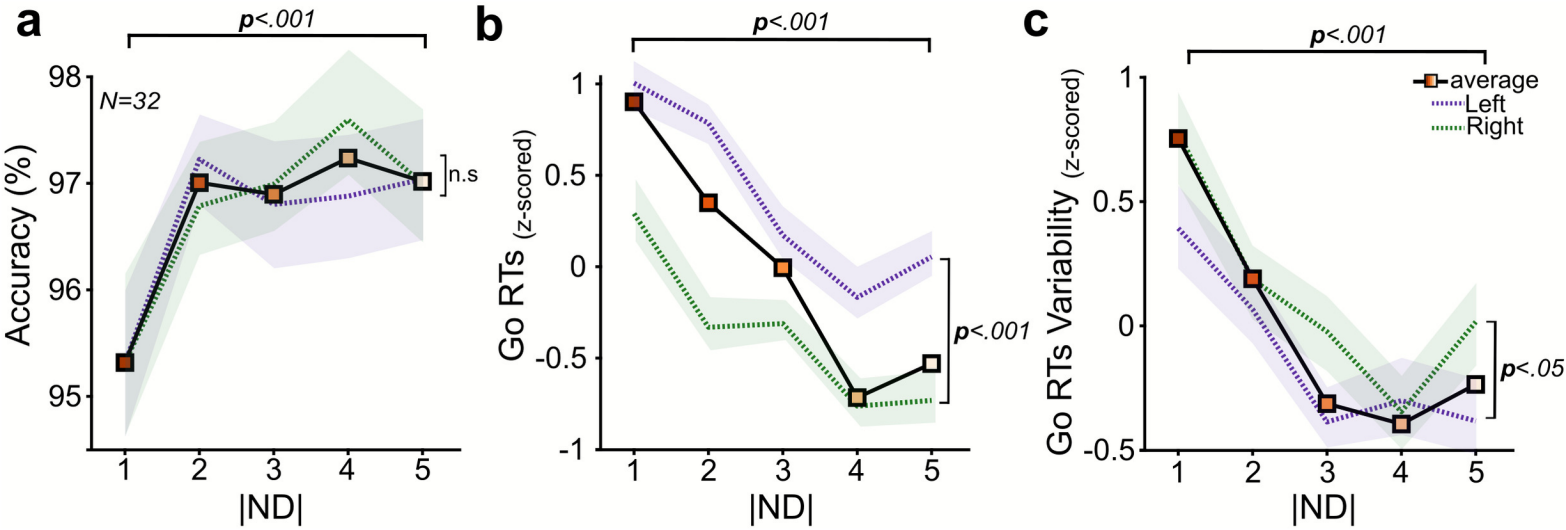
**Performance in Go trials. (a)** Accuracy, **(b)** Go RTs, and **(c)** Go RTs Variability (both z‐scored for visualization purposes only). The analysis considered the representation of both the Left (dashed purple line) and Right (dashed green line) spatial location (SL) of the Go signal presentation. Shaded areas are ±1 SEM; average = averaged across SL.

We assessed the influence of spatial location (SL; Left/Right) and ND (1–5) during movement initiation on Accuracy (Figure 2a) and found a significant increase with increasing ND (two‐way repeated measures ANOVA; *F*(_4,124_) = 7.19, *p* < 0.001) but no significant main effect of the SL (*F*(_1,31_) = 0.13, *p* = 0.71) or significant interaction (*F*(_4,124_) = 0.71, *p* = 0.58). We then examined whether Go RTs and their Variability were influenced by SL and ND (Figure 2b,c).

Go RTs were significantly faster if the higher number was shown on the Right (Right = 540 ms vs. Left = 557 ms) (two‐way repeated measures ANOVA; *F*(_1,31_) = 19.50, *p* < 0.001) and decreased (ND1 562 ± 85 ms; ND2:553 ± 79 ms; ND3:547 ± 79 ms; ND4:540 ± 77 ms; ND5:541 ± 76 ms) as comparison became easier (*F*(_4,124_) = 25.76, *p* < 0.001), with no significant interaction (*F*(_4,124_) = 2.38, *p* = 0.05). Additionally, RTs Variability was higher when the higher number was displayed on the Right (Right = 109 vs. Left = 105) (two‐way repeated measures ANOVA; *F*(_1,31_) = 4.26, *p* = 0.04), but decreased (ND1:116 ± 22 ms; ND2:110 ± 24 ms; ND3:105 ± 23 ms; ND4:105 ± 22 ms; ND5:105 ± 22 ms) significantly as NDs increased (*F*(_4,124_) = 11.87, *p* < 0.001), with no significant interaction found (*F*(_4,124_) = 0.97, *p* = 0.42). Our findings suggest that ND influences movement initiations during Go trials, resulting in increased Accuracy with faster and more consistent RTs when executing movements signaled by the higher number. Additionally, RTs were faster but more variable when a higher number was displayed on the right side.

### 3.2 The Difficulty in Properly Inhibiting a Movement Was Influenced by NDs and SSDs

To evaluate the effect of ND during Stop trials on the ability to inhibit a movement, we first needed to assess whether participants respected the race model's independence assumptions, as violations would preclude a reliable estimation of the SSRT. We used paired *t*‐tests to assess context independence at the group level by comparing Stop Error RTs with Go RTs for each ND (Table 1).

**TABLE 1:** Assessment of context independence across NDs. Average Stop Error RTs and Go RTs for each ND, along with standard deviation and results of paired *t*‐tests comparing the two variables at a population level.

ND	Stop error RT _(ms)_	Go RT _(ms)_	*t‐value*_(31)_	*p*
**1**	550 (±135)	571 (±87)	−0.80	0.43
**2**	531 (±127)	557 (±79)	−0.97	0.33
**3**	498 (±131)	551 (±80)	−1.93	0.06
**4**	490 (±148)	543 (±77)	−1.92	0.06
**5**	483 (±141)	545 (±77)	−2.50	0.01

**FIGURE 3:**
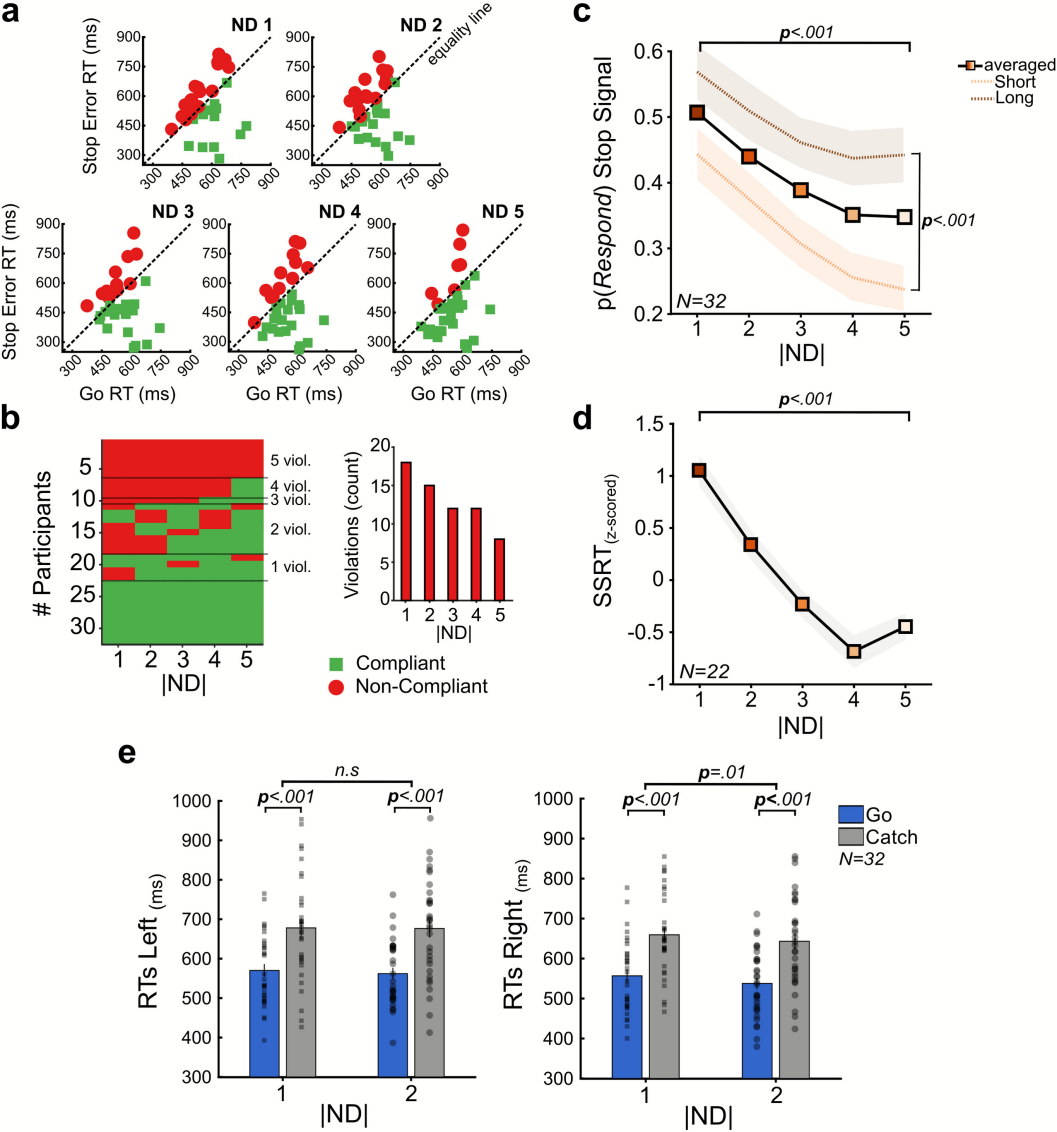
**Assessment of movement inhibition. (a)** Horse‐Race model's assumptions evaluation for each Stop ND. Each participant is represented by a colored marker; green squares indicate a Compliant participant, while red dots indicate Non‐Compliant participants with model assumptions. The equality line is shown for reference. **(b)** Proportion of participants violating model assumptions as a function of ND. The small insert panel reports the absolute number of participants violating the assumptions. **(c)** Influence of ND on p(Respond) to the Stop signal for both short (dashed light orange) and long (dashed brown) SSDs and **(d)** SSRTs (z‐scored for visualization purposes only). **(e)** Catch‐trial slowing is robust and independent of Catch ND. Go‐trial and Catch‐trial RTs for ND1 and ND2, shown separately for Left (left panel) and Right (right panel) responses. Bars indicate the population mean RTs (Go in blue; Catch in gray), and individual participants are overlaid as scatter points. Shaded areas are ±1 SEM; average = averaged across SSDs.

In line with the predictions of the race model, the overall trend indicated that Stop Error RTs were shorter than Go RTs, even though a significant difference was observed only at ND5, indicating that context independence held more consistently for easier comparisons. This pattern suggested that a higher number of violations might be linked to more challenging comparisons. We next assessed context independence at the individual level and observed that as ND increased, the number of participants violating the model's assumption decreased (Figure 3a). We quantified these violations and found that they occurred most frequently at smaller NDs (Figure 3b; small insert panel: ND1 = 18; ND2 = 15; ND3 = 12; ND4 = 12; and ND5 = 8). Given that p(Respond) to the Stop signal quantifying the proportion of trials in which participants failed to inhibit their response provides a model‐free measure of stopping performance, we examined this metric across NDs and SSDs (Figure 3c). Results revealed significant main effects of SSD (two‐way repeated ANOVA; *F*(_1,31_) = 46.42, *p* < 0.001) and ND (*F*(_4,124_) = 23.73, *p* < 0.001), indicating that stopping was less successful at longer SSDs and lower ND. No significant interaction was observed (*F*(_4,124_) = 2.27, *p* = 0.06).

Finally, we estimated the SSRT for a subset of participants who largely complied with the model's assumptions. We applied a threshold to define this subgroup, and participants were excluded if they violated the independence assumption in more than 3ND. This resulted in a subgroup of Non‐Compliant participants (*N* = 10) and the estimation of SSRT for a Compliant group (*N* = 22) that did not exhibit a consistent pattern of violations across ND (Figure 3d). The analysis revealed significant main effects of ND (one‐way repeated ANOVA *F*(_4,84_) = 18.17, *p* < 0.001). SSRT was highest at ND1 and decreased progressively across NDs (ND1: 335 ± 87 ms; ND2: 306 ± 82 ms; ND3: 289 ± 75 ms; ND4: 265 ± 93 ms; ND5: 280 ± 86 ms), with the lowest SSRT values at larger NDs, indicating improved stopping performance as ND increased. To determine whether the transient inhibitory response triggered by the Catch signal also depended on ND, we examined Catch‐trial slowing for the two ND levels in which Catch trials were included (ND1‐2; Figure 3e). We performed two repeated measures ANOVAs, one for Left responses and one for Right responses, with ND (1, 2) and Trial Type (Go, Catch) as within‐subject factors. Both analyses revealed a strong main effect of Trial Type, with Catch trials producing longer RTs than Go trials (Left: *F*(_1,31_) = 31.51, *p* < 0.001; Right: *F*(_1,31_) = 33.66, *p* < 0.001), consistent with a transient, nonspecific pausing response triggered by the Catch signal. The ND × Trial Type interaction was also nonsignificant for both response sides (Left: *F*(_1,31_) = 0.24, *p* = 0.62; Right: *F*(_1,31_) = 0.05, *p* = 0.81), indicating that the magnitude of the slowing effect did not vary with ND. Catch RTs were reliably slower than Go RTs at matched ND levels (Left ΔRT: ND1 = 107 ± 21 ms; ND2 = 102 ± 19 ms; Right ΔRT: ND1 = 114 ± 20 ms; ND2 = 105 ± 18 ms). These findings indicate that ND difficulty modulates both stopping performance and the validity of the race model assumptions, whereas the transient slowing elicited by Catch signals remains stable across ND levels.

### 3.3 Go Drift Rates Underlie Go RT Differences and Predict Successful Stopping Performance

**FIGURE 4:**
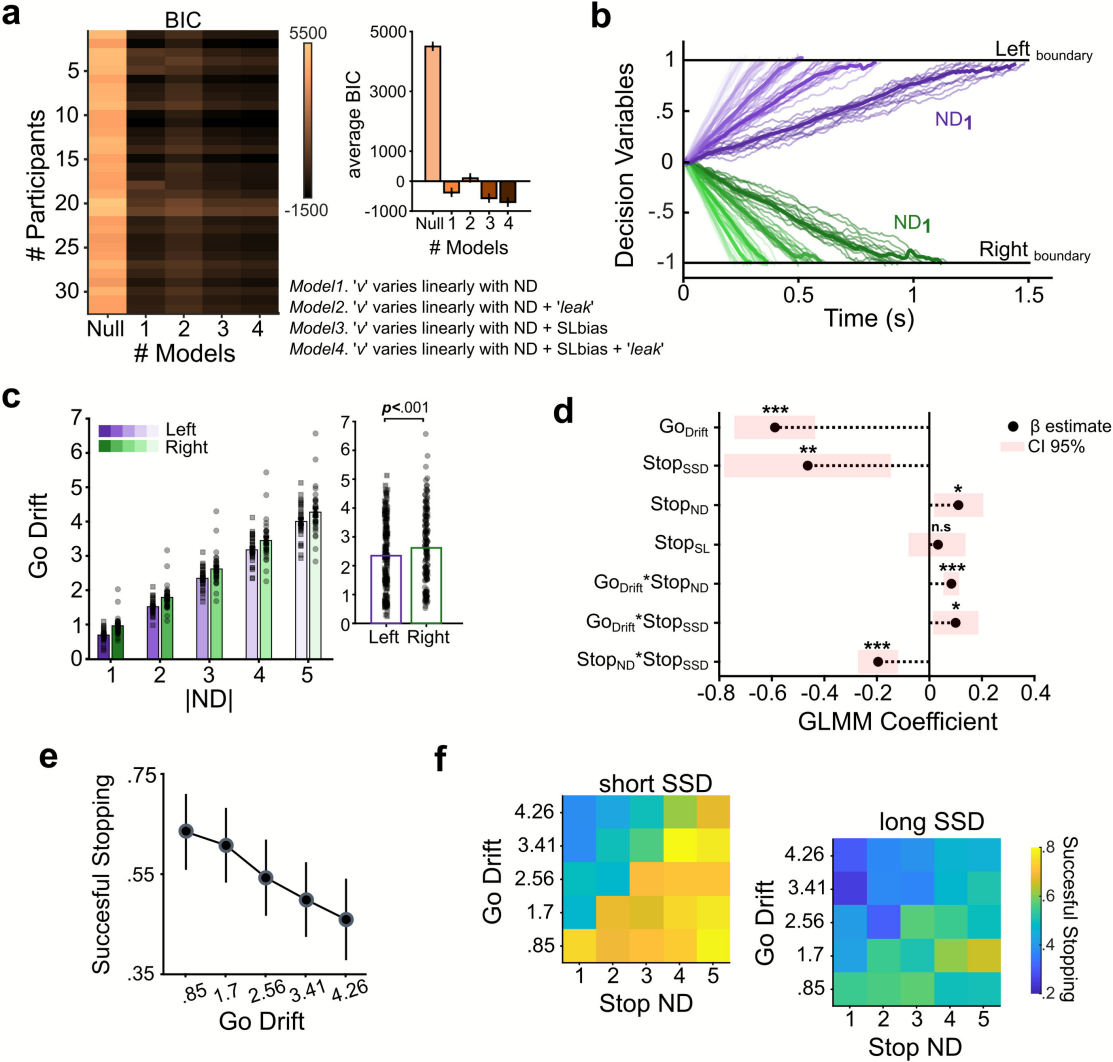
**Effects explained with the GDDM and GLMM. (a)** BIC values for each fitted GDDM model (from Null to Model 4), with each row representing a participant and an insert panel showing group averages ±1 SEM. Model 4 shows the best fit overall. **(b)** Simulated trajectories from the GDDM for a representative participant, using the Go Drift rates from the best‐fitting model. Each colored trace represents a single simulated trial under a given ND and SL (Left = purple shades, Right = green shades). Darker solid lines show the average across trials; black horizontal lines mark decision boundaries. **(c)** Bar plot showing average Go Drifts by ND and SL. The small insert panel shows the significant overall difference between Left and Right SL, with generally higher Go Drift rates for the Right SL, suggesting lateralization in processing speed. **(d)** GLMM coefficient β estimate with a 95% confidence interval. **(e)** Average successful stopping probability decreases as the Go Drift rate increases; higher drift rates triggered by the Go signal with larger ND values correspond to lower successful stopping probability. Error bars represent ±1 SEM. **(f)** Heatmaps showing successful stopping probability as a function of Go Drift (y‐axis) and Stop ND (x‐axis), separately for short SSD (left) and long SSD (right). The effect of Go Drift on successful stopping is mitigated by Stop ND: higher Stop NDs lead to increased stopping probability, whereas lower Stop ND distances reduce stopping probability. Additionally, shorter SSDs (left panel) show generally higher stopping probabilities than longer SSDs (right panel), illustrating the impact of SSD on inhibition success. Color bar indicates the successful stopping probability. Asterisks indicate significance (* < 0.05, ** < 0.01, *** < 0.001).

To assess GDDM model performance, we used the Bayesian information criterion (BIC). Among the candidate models, we found that Model 4 provided the best‐fitting model to the data (Figure 4a), indicating that decision‐making performance was best explained by variations in Go drift rates across ND, SL bias, and the leak term capturing imperfect evidence integration. In addition to yielding the lowest BIC, Model 4 also had the highest (least negative) log‐likelihood, confirming that its superior fit was not simply due to a higher number of parameters. Average parameter estimates are reported in Table 2.

**TABLE 2:** Average fitted parameters across participants for each model by the GDDM. Model 1 *Linear*; Model 2 *LinearwithLeak*; Model 3 *LinearwithSLbias*; and Model 4 *LinearwithSLbiasLeak*.

Model	Drift	SL*bias*	Leak	Nondecision (ms)	BIC
**1**	0.80 (±0.11)	—	—	317 (±79)	−415.7 (±567.8)
**2**	−0.22 (±0.31)	—	9.05 (±1.39)	231 (±85)	+32.4 (±408.1)
**3**	0.84 (±0.10)	−0.08 (±0.13)	—	314 (±79)	−599.7 (±442.1)
**4**	0.91 (±0.14)	−0.15 (±0.22)	6.34 (±1.82)	232 (±81)	−766.4 (±448.8)

To visualize these dynamics, Figure 4b shows simulated decision trajectories generated using Go Drift rates from the best‐fitting model for a representative participant, highlighting the influence of both ND and SL bias.

The trajectories show faster evidence accumulation toward the lower Right boundary, reflecting the rightward SL bias (−0.13 ± 0.20), while accumulation toward the upper Left boundary is comparatively slower. Analysis of the fitted Go Drift rates revealed an increase with ND (ND1 = 0.82 ± 0.11; ND2 = 1.65 ± 0.22; ND3 = 2.48 ± 0.34; ND4 = 3.31 ± 0.45; ND5 = 4.13 ± 0.57), showing faster evidence accumulation for larger NDs. Furthermore, there were significantly higher Go Drifts toward the Right SL (small insert panel; paired *t*‐test: ***t***_**(159)**_ = −8.48, *p* < 0.001) (Figure 4c).

Building on these findings, we next examined whether Go Drift rates influenced trial‐wise stopping performance. Using a GLMM, we predicted the probability of successful stopping based on parameters derived from Stop trials (see Section 2 for details and Figure 4d). The analysis revealed that higher Go Drift values were significantly associated with lower stopping success (β_1_ = −0.59, *p* < 0.001), supporting the hypothesis that stronger Go‐related evidence accumulation impairs inhibition. This relationship was also observed in the empirical data, where higher Go Drift corresponded with lower stopping success (Figure 4e). As expected, longer SSDs were linked to decreased stopping success (β_2_ = −0.46, *p* = 0.004), while greater Stop ND predicted improved stopping performance (β_3_ = +0.11, *p* = 0.02). This suggests that increased Stop ND facilitates inhibitory control. In contrast, Stop SL showed no significant effect after controlling for other predictors (β_4_ = +0.03, *p* = 0.60). We also observed a significant interaction between Go Drift and SSD (β_5_ = +0.10, *p* = 0.02), indicating that Stop trials with both high Go Drift and long SSDs were associated with particularly poor stopping performance. However, this detrimental effect of Go Drift was mitigated when Stop ND was high, as shown by a significant Go Drift × Stop ND interaction (β_6_ = +0.08, *p* < 0.001). A further interaction between Stop ND and SSD (β_7_ = −0.20, *p* < 0.001) revealed that the benefits of high Stop ND were especially pronounced at shorter SSDs (Figure 4f). Overall, these results indicate that stopping success depends not only on the individual properties of Go and Stop processes but also on their interactions. Specifically, inhibitory control is shaped by the strength of Go evidence (Go Drift), the temporal window for inhibition (SSD), and the distinctiveness of the Stop ND, all of which jointly determine stopping efficiency on a trial‐by‐trial basis.

## 4 Discussion

Our study shows that both movement initiation and inhibition were influenced by the cognitive difficulty in interpreting the information provided by the comparison of numbers used as stimuli. Different levels of cognitive difficulty were introduced by variations in the ND, for both the Go and the Stop signal, even though the visual appearance of the stimuli remained perceptually unambiguous across trials. Our results suggest that motor decisions are influenced by cognitive processing, not only by stimulus‐driven visuomotor transformations, offering new insights into how decision‐making integrates both cognitive and perceptual content. Previous studies focused primarily on how perceptual difficulty influences motor performance during Go trials. Pressing buttons or saccadic responses have been prompted by the spatial location of visual stimuli (Logan 1981; Logan and Irwin 2000) or by prompting movements on the base of the visual similarity of alphabetic letters (Osman et al. 1986), with varying degrees of distinguishability, such as “G vs. X” or “I vs. i” for relatively easy or difficult comparisons, showing that increased ambiguity increases the latency of the responses. Middlebrooks and Schall (2013) similarly varied the proportion of colors in a checkerboard search array that cued leftward or rightward saccades, so altering target discriminability. They found that saccade latency decreased as color dominance increased. These results prove that faster movements are facilitated by the easy perceptual process. On the other hand, a different line of research concentrated on how the salience of the Stop signal affects movement inhibition, showing that reducing the perceptual discriminability results in slower inhibition (Van Der Schoot et al. 2005; Morein‐Zamir and Kingstone 2006; Montanari et al. 2017; Middlebrooks et al. 2019). In Montanari et al. (2017), participants initiated reaching movements based on a colored Go signal and stopped them depending on a color change in the Stop signal, with findings indicating that perceptual color similarity between Go and Stop signals increased SSRT. While all these studies underscore the importance of perceptual discriminability in motor control, they primarily address lower‐level sensory factors; our findings expand this framework by focusing on the cognitive informational content, varying the difficulty required to extract meaning from stimuli while maintaining constant perceptual features. In our task, both the Go and Stop signals are tied to a mathematical rule requiring participants to evaluate the numerical relationship between numbers by manipulating the ND, thereby introducing a factor intervening in a phase of stimulus processing after the perceptual encoding, which we show to significantly influence motor decision. In fact, our results (Figure 2) show that when the stimulus‐selective processing engages a higher cognitive difficulty, movement initiation is delayed, as when perceptual ambiguity is higher (Middlebrooks and Schall 2013), but the same factor also influenced the recruitment of the Stop process. These distance‐related effects have been previously observed to influence motor decisions (Acuna 2002; Brunamonti et al. 2012, 2016; Krajcsi et al. 2016; Mione et al. 2020; Ramawat et al. 2022, 2023; for a review), and here, we provide evidence that they also intervene in modulating inhibitory control. Specifically, participants' performance during movement initiation showed improved Go accuracy and faster RTs at larger NDs, indicating that more distinct numerical contrasts reduce cognitive demand and facilitate decision‐making. Furthermore, the hypothesis of a spatially oriented representation was supported by the fact that RTs were modulated by the SL of the Go signals so that when higher numbers appeared on the right side, responses were faster than when they appeared on the Left. Similarly, ND also modulated movement inhibition in Stop trials (Figure 3). Within the SST framework, which models movement inhibition as the outcome of a race between independent Go and Stop processes (Logan and Cowan 1984), we observed that p(Respond) depends on the time the Stop process is recruited and how far the Go process is from the movement's onset threshold. As typically described, even here, we observed that SSD is a factor influencing motor inhibition; in addition, here, we provide evidence that variables interact with the difficulty in engaging the Stop process as a function of the Stop ND. It is likely that the time required to process closer numerical values delayed the initiation or progression of the Stop process, delaying the SSRT, which we observed to be longer for smaller ND, allowing the Go response to reach threshold. This elongation of SSRT at small ND suggests that numerical comparison difficulty may influence both the onset and the subsequent rate of rise of the stopping process. Because our data cannot determine whether ND primarily delays the onset of stopping or slows the inhibitory rise (or both), we interpret this effect within a broad multistage framework encompassing models that separate stimulus evaluation from inhibitory engagement (Logan et al. 2014) as well as models proposing sequential pausing and cancellation mechanisms (Diesburg and Wessel 2021; Wadsley et al. 2023). In addition, more participants failed to meet the model's independence assumption criteria at smaller Stop NDs, indicating that violations were themselves ND‐dependent and more common under higher cognitive difficulty. Although the independence assumption is typically met in classical SST studies (Verbruggen and Logan 2009), previous research has shown that dependence between Go and Stop processes can emerge in selective SST variants, especially when response selection becomes more complex (De Jong et al. 1995; Bissett and Logan 2014; Giarrocco et al. 2021). Verbruggen and Logan (2015) similarly reported increased dependence when the Stop signal was harder to discriminate or interpret. By comparing mean failed Stop RTs with mean Go RTs, they found that, under higher signal ambiguity (varied‐mapping conditions), failed Stop RTs were longer than Go RTs, indicating a violation of independence. In contrast, failed Stop RTs remained shorter in low‐ambiguity (consistent‐mapping conditions), consistent with race model predictions. Our results align with this view, showing that increased decision difficulty at smaller NDs was associated with greater dependence between processes, as reflected in a higher number of participants exhibiting longer mean Stop Error RTs than mean Go RTs. A further variable influencing the model's compliance is the structure of the task. The inclusion of Catch signals requires participants to discriminate between Stop and “ignore” stimuli before executing or withholding an action. As noted by Bissett and Logan (2014), this additional discrimination step can impose extra processing demands on the Go process and thereby lengthen Go RTs. Such elongation introduces potential challenges for SSRT estimation in stimulus‐selective SSTs, since the independence assumption of the horse‐race model may be partially compromised when Go RTs are influenced by contextual uncertainty or strategy adjustments. This lengthening of Go RTs suggests that SSRT estimates in selective stopping paradigms should be interpreted with caution. In addition to these considerations, our direct analysis of Catch trials (Figure 3e) provides further clarification of the mechanisms underlying the observed effects. Catch signals reliably produced longer RT than Go trials, consistent with a rapid, nonspecific “pause” response described in the pause‐then‐cancel framework (Diesburg and Wessel 2021; Giarrocco et al. 2021) and with recent demonstrations that selective stopping elicits an early, global suppression of motor output before more selective inhibitory processes emerge (Wadsley et al. 2023). Crucially, this Catch‐induced slowing was stable across the two ND levels in which Catch trials occurred, indicating that attentional capture or stimulus‐driven interference did not scale with ND. Therefore, the ND‐dependent variations we observed in Go RTs and SSRT are unlikely to arise from differential distraction elicited by Catch stimuli and instead reflect the difficulty of evaluating rule‐relevant numerical information.

Extending our behavioral findings, the modeling results provided deeper insights into how cognitive interpretability shapes motor decision‐making during movement initiation (Figure 4b,c). Using the GDDM, which decomposes the decision process by isolating key parameters such as drift rate, decision boundary, and starting point, we were able to identify the latent cognitive parameters that underlie movement initiation during Go trials. In our task design, we assumed a fixed starting point due to the simple button‐release response mapping, which enabled us to control for internal factors like reward expectancy or motivation that are known to reduce the distance between the starting point and the decision threshold when engaged (Ratcliff 1985; Diederich and Busemeyer 2006; Giuffrida et al. 2023). Similar to this, we assumed stable decision boundaries because the task did not include explicit speed‐accuracy trade‐offs instructions or manipulations of perceptual uncertainty, reducing the likelihood of systematic variations in response caution or strategy across NDs (Ratcliff et al. 2015). This does not preclude the presence of general proactive adjustments typical of SST (e.g., proactive slowing) but rather indicates that they were not experimentally manipulated in our design. Consistent with this, the best‐fitting model (Model 4) among the tested models had ND‐modulated Go Drifts, an SL bias, and a leak parameter that showed imperfect integration over time. This result is consistent with classical effects that indicate slower and more error‐prone comparisons are produced by lower ND (Moyer and Landauer 1967; Krajcsi et al. 2016). This suggests that the drift rate mainly reflects cognitive demand. In parallel, the spatial configuration of the response targets introduced an SL bias. Specifically, trials where the higher number appeared on the right side consistently produced higher drift rates, suggesting a spatial advantage. This SL bias, consistent with the MNL, proposes a left‐to‐right spatial mapping of numerical values and is consistent with prior findings on spatial‐numerical associations influencing movement selection and perceptual judgments (Dehaene et al. 1993; Umiltà et al. 2009). In our final analysis, using a GLMM analysis, we examined the influence of previously analyzed variables on successful movement inhibition. We observed that a higher Go Drift (i.e., faster evidence accumulation in the Go process) had the strongest influence among the variables analyzed, consistent with findings that higher response preparation can facilitate movement initiation but hinder motor control (Andujar et al. 2022). The second most influential variable in action stopping was the duration of the SSD. Both variables, whether considered individually or in interaction, align with the core predictions of the race model (Logan and Cowan 1984). The GLMM also indicated that other variables, such as Stop ND, modulated these effects, particularly when the Stop signal was easy to decode. In such cases, longer SSDs amplified the impact of high Go Drifts, while higher Stop ND mitigated it, increasing the likelihood of inhibition.

These findings indicate that movement inhibition depends not only on the timing of the Stop signal but also on how effectively participants extract and process relevant information content from both Go and Stop signals. At the single‐trial level, the probability of interrupting an undesired movement reflects the configuration of multiple interacting variables. Specifically, successful movement inhibition is more likely when the Go process has a lower Go Drift rate, the Stop signal appears early (short SSD), and the Stop signal is easily encoded (high Stop ND). On the contrary, inhibition is impaired when these variables assume opposite values. Overall, our results underscore the importance of cognitive variables involved in processing the Stop signal and how their interaction with core SST variables, such as Go RT and SSD (Logan and Cowan 1984), shapes movement inhibition. These findings are consistent with prior work emphasizing the role of intervening variables in shaping the race dynamics between Go and Stop processes, such as motivational (Giuffrida et al. 2023, 2025), perceptual (Middlebrooks and Schall 2013), attentional (Haque et al. 2024), and even biomechanical variables (Ramawat et al. 2024; Fiori et al. 2025). For example, the latter studies highlighted the need to include biomechanical variables, such as baseline pre‐action force (Ramawat et al. 2024) and center of body mass displacement when the whole body is engaged in movement (Fiori et al. 2025), in the models developed to investigate movement generation and inhibition. The integration of these variables would provide contexts where studying action control better fits the complexity of a realistic framework that simulates real‐life scenarios. The present results extend the set of variables that should be considered when applying the SST to study motor inhibition across different contexts. Here, we provide evidence that proper movement control may depend on the difficulty of applying a rule to determine whether to stop or continue an engaged action.

## Author Contributions

Emiliano Brunamonti supervised the overall project. Emiliano Brunamonti, Stefano Ferraina, and Sabrina Fagioli provided key resources and research infrastructure. Emiliano Brunamonti and Isabel Beatrice Marc conceived and designed the study, carried out the investigation, and contributed to data interpretation. Isabel Beatrice Marc developed the methodology, performed formal analysis, and prepared the visualizations and figures. Isabel Beatrice Marc and Valentina Giuffrida conducted the modeling and simulation work. Mariella Segreti and Ann Paul were responsible for data acquisition, curation, and recruitment of participants for the study. Emiliano Brunamonti, Isabel Beatrice Marc, and Valentina Giuffrida wrote the original draft of the manuscript. All authors reviewed, edited, and approved the final version of the manuscript.

## Ethics Statement

This study was approved by the ethics committee at University of Roma Tre.

## Conflicts of Interest

The authors declare no conflicts of interest.

## Data Availability

The data on which this paper is based will be made available upon reasonable request to the corresponding author.
